# Experimentally Induced Hyperinsulinemia Fails to Induce Polycystic Ovary Syndrome-like Traits in Female Rhesus Macaques

**DOI:** 10.3390/ijms23052635

**Published:** 2022-02-27

**Authors:** Rao Zhou, Cristin M. Bruns, Ian M. Bird, Joseph W. Kemnitz, Daniel A. Dumesic, David H. Abbott

**Affiliations:** 1 Wisconsin National Primate Research Center, University of Wisconsin, Madison, WI 53715, USA; raozhou06@yahoo.com (R.Z.); kemnitz@primate.wisc.edu (J.W.K.); 2 Endocrinology Reproductive Physiology Training Program, University of Wisconsin, Madison, WI 53715, USA; imbird@wisc.edu; 3 Departments of Medicine, University of Wisconsin, Madison, WI 53715, USA; bruns@oklahomaheart.com; 4 Departments of Obstetrics and Gynecology, University of Wisconsin, Madison, WI 53715, USA; 5 Departments of Cell and Regenerative Biology, University of Wisconsin, Madison, WI 53715, USA; 6 Department of Obstetrics and Gynecology, David Geffen School of Medicine, University of California, Los Angeles, CA 90095, USA; ddumesic@mednet.ucla.edu

**Keywords:** developmental programming, testosterone, prenatally androgenized, ovarian hyperandrogenism, non-human primate model, oligomenorrhea

## Abstract

As in women with polycystic ovary syndrome (PCOS), hyperinsulinemia is associated with anovulation in PCOS-like female rhesus monkeys. Insulin sensitizers ameliorate hyperinsulinemia and stimulate ovulatory menstrual cycles in PCOS-like monkeys. To determine whether hyperinsulinemia (>694 pmol/L), alone, induces PCOS-like traits, five PCOS-like female rhesus monkeys with minimal PCOS-like traits, and four control females of similar mid-to-late reproductive years and body mass index, received daily subcutaneous injections of recombinant human insulin or diluent for 6–7 months. A cross-over experimental design enabled use of the same monkeys in each treatment phase. Insulin treatment unexpectedly normalized follicular phase duration in PCOS-like, but not control, females. In response to an intramuscular injection of 200 IU hCG, neither prenatally androgenized nor control females demonstrated ovarian hyperandrogenic responses while receiving insulin. An intravenous GnRH (100 ng/kg) injection also did not reveal evidence of hypergonadotropism. Taken together, these results suggest that experimentally induced adult hyperinsulinemia, alone, is insufficient to induce PCOS-like traits in female rhesus monkeys and to amplify intrinsic PCOS-like pathophysiology.

## 1. Introduction

Polycystic ovary syndrome (PCOS) is the most common endocrinopathy underlying anovulatory infertility in reproductive-aged women [1,2], and is characterized by ovarian hyperandrogenism, oligo-anovulation, and polycystic ovaries [3,4,5]. Endogenous hyperinsulinemia, found in most women with PCOS [3,5,6,7,8,9] with or without obesity, has been confirmed in several studies [9,10,11,12,13] and is associated with hyperandrogenic anovulation [7,14]. Compensatory pancreatic hyperinsulinemia, due to insulin resistance in metabolic target tissues (i.e., adipose, skeletal muscle and liver), enhances PCOS phenotypic expression [9,15,16,17,18] and pathogenic metabolic sequelae [13]. In PCOS women, moreover, diminished insulin-mediated glucose uptake in ovarian [19] and non-ovarian organ tissues [7,9,20], is accompanied by normal ovarian steroidogenic responsiveness to insulin [21,22,23], possibly through an alternative inositolglycan signal transduction system [24].

Clinical studies of obese women with PCOS, employing weight loss programs [25,26,27], bariatric surgery [28], insulin-sensitizing agents in obese and non-obese patients [24,29,30,31,32], anti-androgens [33,34] and mental health intervention [35], have diminished insulin resistance and improved ovulatory function. Such weight loss strategies, however, while reducing hyperinsulinemia from insulin resistance [25,26,27], also alter metabolic and adipocyte functions through leptin and/or adiponectin production [36,37], with subsequent actions on ovarian steroidogenesis [35,38] and/or folliculogenesis [39]. Insulin-sensitizing therapies can also have additional direct effects on ovarian function [22,40,41,42,43,44,45]. Thiazolidinediones inhibit androgen while stimulating progesterone production in human ovarian cultures in the absence of insulin exposure [46,47]. The commonly used insulin-sensitizer and anti-gluconeogenic agent metformin [31,48] inhibits steroidogenesis in both human granulosa and theca cells in vitro [41,49,50,51], while inositols enhance follicle-stimulating hormone (FSH)-stimulated estradiol release from ovarian granulosa cells and reduce circulating testosterone levels [42,43,44,45]. Thus, while inducing ovulation in PCOS women, insulin-reducing therapies have additional effects on ovarian function that may benefit induction of ovulatory menstrual cycles [52,53]. Taken together, these findings from women with PCOS provide circumstantial evidence that implicate hyperinsulinemia from insulin resistance with PCOS traits, including hyperandrogenic anovulation.

Due to the risks of hypoglycemia and weight gain, no studies have investigated the hypotheses that experimentally controlled, long-term exogenous insulin treatment engenders PCOS in healthy women, and/or amplifies the severity of PCOS phenotypic expression. Intensive insulin therapy, nevertheless, is essential for clinical management of women with type 1 diabetes (T1D) who are insulin sensitive [54], and for women with T2D who have pronounced insulin resistance [55]. Twenty-four percent of women with T1D exhibit PCOS [56], comparable to PCOS incidence in women with T2D [57]. Prevalence of PCOS in women with diabetes is thus notably higher than PCOS prevalence in women without diabetes [39,52,56]. In addition, women with T1D or T2D exhibit a higher than typical incidence of hyperandrogenism (25%, 20%, respectively, compared to healthy women) and irregular menstrual cycles (24%, 18%), as key diagnostic criteria for PCOS [57], thus implicating exogenous insulin therapy in the pathogenesis of PCOS [39]. Exogenous insulin therapy, nevertheless, is associated with PCOS in a minority of women with either T1D or T2D [39,57,58]. In women with T1D, moreover, signs and symptoms of androgen excess are milder than would be typical for women with PCOS, while circulating sex hormone-binding globulin (SHBG) concentrations are normal, instead of being reduced as in PCOS [58,59], suggesting additional factor(s) separate from hyperinsulinemia as key to PCOS pathogenesis [13,39,60].

Adiposity-associated hyperinsulinemia is linked with an ~80% incidence of anovulation in adult PCOS-like female rhesus monkeys [61] that were previously exposed to early- to mid-gestational testosterone excess. Conversely, PCOS-like female monkeys with normal circulating insulin levels exhibit only 21% incidence of anovulation [61], outside summer months of oligomenorrhea in this seasonally breeding species [62]. Such an association between elevated circulating insulin levels and anovulation is of particular note, since PCOS-like monkeys exhibit many of the reproductive and metabolic defects found in women with PCOS [63,64], implying a fetal origin for the syndrome [65,66]. Adult female rhesus monkeys, with highly comparable reproductive and metabolic physiology and pathophysiology to women, can exhibit naturally occurring T1D [67,68], T2D [69] and PCOS-like traits [70], as well as demonstrating gestational testosterone induction of PCOS-like traits in female monkeys [61], thus providing unparalleled animal models to functionally test whether administration of exogenous insulin, alone, can induce PCOS-like traits.

Equally important, the risk of inducing insulin-mediated hypoglycemia in PCOS-like and control (healthy, regularly cycling) monkeys with intact counter-regulatory mechanisms (i.e., glucagon, epinephrine and glucocorticoids) is minimal because maintaining monkeys in stable, social and physical environments, with controlled, routine provision of a balanced diet, allows progressive increases in insulin amounts injected daily, together with twice-daily monitoring of blood glucose concentrations to reduce the risk of hypoglycemia from exogenous insulin administration [71,72,73], as has been reported for humans with T2D [55,74]. Thus, in this cross-over designed study, in which the same monkeys were employed in both arms of this study, we administered daily insulin or placebo subcutaneous injections to both control and PCOS-like female monkeys for 6 to 7 months to experimentally increase circulating insulin (1) to levels that match or exceed those levels associated with anovulation in PCOS-like monkeys [61]; (2) potentially inducing ovarian hyperandrogenism, hypergonadotropism and weight gain; (3) impairing ovulatory function; and (4) replicating a PCOS-like phenotype in female non-human primates [63]. To detect these reproductive impairments, only PCOS-like female monkeys that exhibited minimal menstrual cycle and metabolic PCOS-like traits were selected for study. Such selection criteria were expected to generate a PCOS-like monkey group that exhibited minimal adult androgenic and LH excess, similar to ovulatory women with PCOS [13,75,76], prior to chronic insulin treatment. All monkeys underwent the same series of reproductive and metabolic observations and challenge tests within each arm of this study.

## 2. Results

### 2.1. Metabolic Observations

#### Glucoregulatory Effects of Chronic Insulin Therapy

Morning fasting circulating levels of insulin first exceeded the target level of 694.5 pmol/L after 62.0 ± 27.9 days (mean ± SEM; ~2 months) and 26.8 ± 12.0 days (~1 month) in control and PCOS-like female monkeys, respectively (*p* ≤ 0.25). Attaining this target circulating insulin level required similar doses of humulin U ultralente in both female groups (control: 9.0 ± 1.5 U/day, PA: 7.8 ± 1.7 U/day; *p* ≤ 0.63). These daily doses of humulin U ultralente resemble those given in partial insulin treatment of naturally occurring T2D in adult rhesus monkeys at Wisconsin National Primate Research Center (WNPRC) (6–20 U/day), and these diabetic animals also received the same daily dose of intermediate-acting human insulin (NPH) [77], and both insulin preparations and diluent were clinically approved for use when this study was undertaken. As illustrated in Table 1, fasting serum insulin levels were increased (*p* < 0.01) by daily insulin injections, reaching median levels of 1219 and 745 pmol/L in insulin-treated control and PCOS-like monkeys, respectively, during the 6 month insulin treatment phase, compared to a 6 month median of 509 and 364 pmol/L in the diluent treatment phase, respectively, in the same female groups. Only gradual alterations in insulin doses were required to maintain target insulin elevations across the 6–7 month treatment phase, with insulin doses at the end of the treatment period either minimally changed (control females: 8.8 ± 2.2 U/day) or increased by ~50% (PCOS-like females: 12.2 ± 1.2 U/day), the latter suggesting a more pronounced degree of insulin resistance in PCOS-like female monkeys. There were no significant variations in circulating insulin levels in either female group once the target circulating level was exceeded. As indicated in Table 1, circulating insulin levels in females receiving insulin exceeded those for females receiving placebo injections in both female groups between months 2–6 of treatment.

In examining the acute increase in circulating insulin levels following daily insulin injections (during the 3rd–5th 30 day period or menstrual cycle at approximately 3–5 months of treatment), circulating insulin levels rose (*p* ≤ 0.02, Bonferroni corrected for six comparisons) above morning basal levels by ~364% and ~32% in control and PCOS-like females, respectively (Table 2), 4 h after daily insulin injections. The insulin injections induced a greater (*p* < 0.01) daily rise in circulating insulin levels in both female groups compared to those observed following diluent injections (Table 2).

Not surprisingly, while receiving daily insulin injections, fasted morning glucose levels were reduced (*p* < 0.04) by ~5.6% and ~4.9% in control and PCOS-like females, respectively, as well as an afternoon glucose decrease of 3.8% in controls and of 11.9% in PCOS-like monkeys compared to values achieved when the same monkeys received diluent injections (Table 1). Mean blood glucose values during insulin therapy, nevertheless, were 3.6 and 4.5 mmol/L in control and PCOS-like monkeys, respectively, in comparison to 3.8 and 5.0 mmol/L, respectively, in the same monkeys while receiving placebo injections, and notably greater than the 50th (3.3 mmol/L) percentile for morning fasted circulating glucose levels found in 134 non-diabetic, adult rhesus monkeys at WNPRC [73].

### 2.2. Metabolic Challenge Tests

#### Frequently Sampled Intravenous Glucose Tolerance Tests (FSIGTT) and Minimal Modeling

Monkeys received an FSIGTT during either the 3rd–4th menstrual cycle or anovulatory 30 day period from treatment onset (at ~3–4 months of treatment). Despite chronic exogenous insulin treatment, there were no significant changes in measures of either insulin sensitivity (S_I_) or glucose clearance (K_G_), as estimated by the Minimal Model of Bergman (Table 3) [78]. As expected, nevertheless, chronic insulin therapy elevated basal insulin levels (*p* ≤ 0.009) and area-under-the-curve insulin values between 0 and 180 min following glucose infusion (AUC_INS(0–180 min)_) values (*p* ≤ 0.04) above those found during diluent treatment in both female groups (Table 3). Despite exogenous insulin treatment, basal C-peptide values and glucose- and tolbutamide-stimulated C-peptide AUCs were unaltered (*p*-values all ≤ 0.18) in both control and PCOS-like female monkeys, suggesting a lack of suppression of endogenous beta cell insulin secretion induced by exogenous insulin excess.

Despite a slight reduction in morning fasted glucose levels induced by insulin therapy, basal glucose levels and all AUC_GLU_ values during the FSIGTT were unaffected (Table 3). Insulin therapy associated decrements (*p* ≤ 0.02) in acute insulin response to glucose (AIR_g_) probably contributed to the accompanying decrease (*p* ≤ 0.02) in disposition index (DI) exhibited by both female groups (Table 3), and both were likely consequences of artificially elevated baseline insulin values due to insulin injections. The lower (*p* ≤ 0.03) AUC for insulin immediately following infusion of glucose (AUC_INS (0–19)_) found only in PCOS-like monkeys, regardless of treatment, may reflect a previously reported impaired insulin response to glucose in these females indicative of failing pancreatic beta cell compensation [79,80].

Incubation of ^125^I-radiolabeled human insulin with assay buffer, rhesus serum quality control samples, and serum samples from individual PCOS-like and control females 6–7 months after treatment onset revealed little serum binding of insulin above that of assay buffer or rhesus quality control serum. Specific binding ranged from 0 to 2.7% and 0.3 to 24.6% in PCOS-like and control, females, respectively, with only one control female exhibiting serum specific binding of insulin above 3.0%. The latter single control female with notable serum binding capacity for insulin, however, exhibited similar glucoregulatory and reproductive outcomes to the remainder of the control group. Thus, there appeared to be little experimentally induced serum binding of insulin in either PCOS-like or control monkeys during 6–7 months of daily injections with exogenous recombinant human insulin, and thus no obvious evidence for serum neutralization of exogenous insulin treatment.

### 2.3. Body Weights and Blood Chemistry Panels

Body weights did not differ between groups either at the beginning or cessation of treatments (Table 4). Total cholesterol, triglyceride and liver enzyme values were comparable between female groups and across treatments (Table 4) and typical for adult female monkeys [81].

### 2.4. Menstrual Cycle

**Figure 1:**
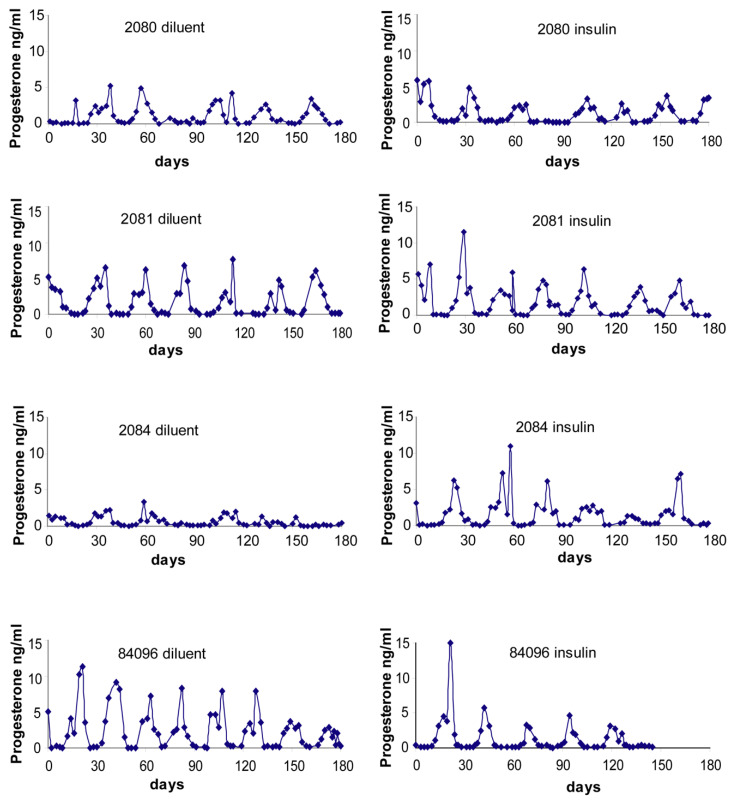
Serum progesterone profiles across menstrual cycles of the four individual control females that underwent both diluent and insulin treatment arms of this study. SI units conversion: progesterone × 3.18 nmol/L.

**Figure 2:**
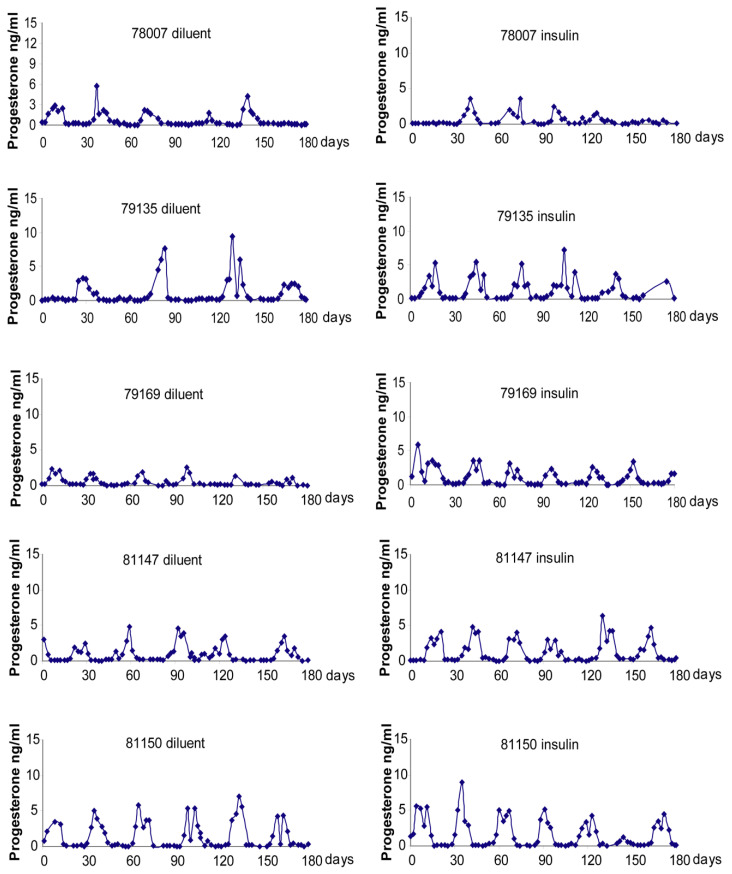
Serum progesterone profiles across menstrual cycles of the five individual PCOS-like female monkeys that underwent both diluent and insulin treatment arms of this study. SI units conversion: progesterone × 3.18 nmol/L.

**Figure 3:**
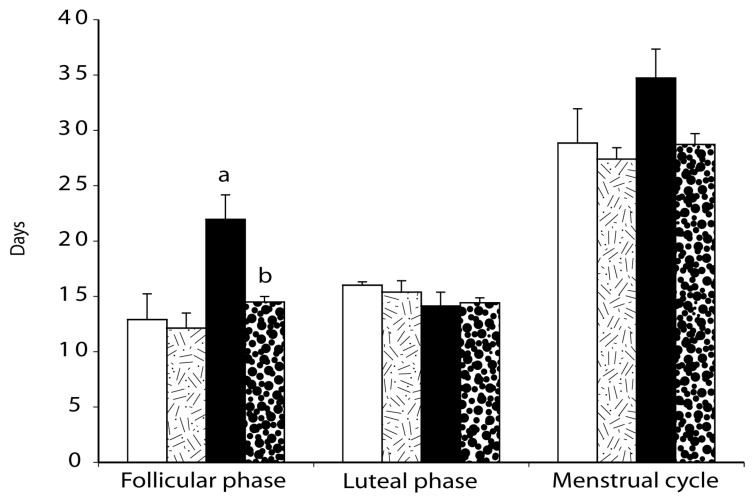
Mean (± SEM) durations of follicular phase, luteal phase and menstrual cycle are illustrated for both control (*n* = 4; diluent: white, insulin: lined) and PCOS-like (*n* = 5; diluent: black, insulin: dotted) female rhesus monkeys. Duration of the follicular phase in PCOS-like monkeys was normalized during insulin treatment. a, *p* < 0.04 vs. control female monkeys receiving diluent; b, *p* < 0.04 vs. PCOS-like female monkeys receiving diluent.

Individual control and PCOS-like monkey menstrual cycle durations and circulating progesterone levels are illustrated in Figure 1 and Figure 2. As illustrated in Figure 3, in PCOS-like female monkeys alone, duration of the follicular phase was diminished on insulin compared to diluent treatment (diluent: 21.9 ± 2.2 days, insulin: 14.5 ± 0.5 days, *p* ≤ 0.02). Follicular phase duration was not changed by insulin treatment in control monkeys (placebo: 12.0 ± 2.3 days, insulin: 12.1 ± 1.4 days, *p* ≤ 0.8). Furthermore, follicular phase duration was longer (*p* ≤ 0.03) in PCOS-like compared to control female monkeys during diluent treatment, but was not different during insulin therapy.

**Figure 4:**
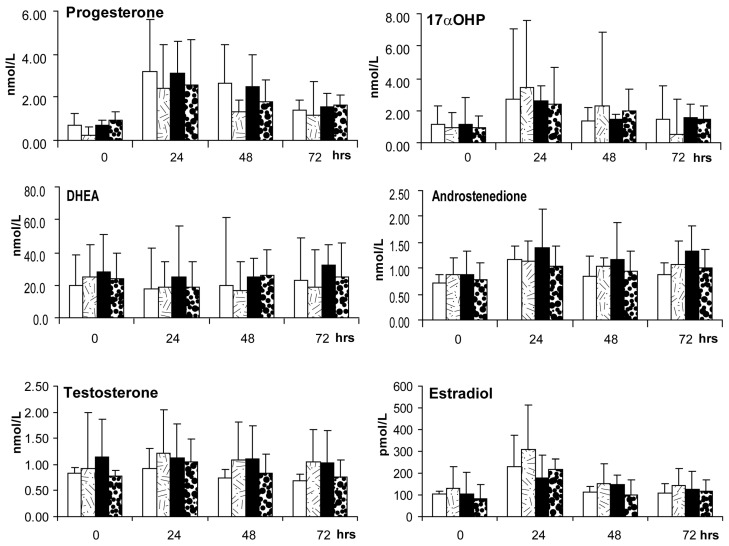
Androgen stimulation test. Serum steroid hormone values (mean ± SEM) after 200 IU recombinant human chorionic gonadotropin (hCG) intramuscular injection at ~3–5 months following treatment onset. Insulin treatment did not increase any steroid hormone response and values did not differ between control and PCOS-like female monkeys. Serum levels of 17α-hydroxyprogesterone (17αOHP), androstenedione, estradiol and progesterone increased (*p*-values < 0.05) following hCG injection, and reached peak levels after 24 h. DHEA and testosterone levels were unresponsive to hCG. Control females: *n* = 4; diluent treatment, white bars; insulin treatment, lined bars, and PCOS-like females: *n* = 5; diluent treatment, black bars; insulin treatment, dotted bars.

In contrast, duration of the luteal phase was not altered (*p* ≤ 0.8) by insulin treatment (diluent: control females, 16.0 ± 0.3 days; PCOS-like, 14.1 ± 1.3 days; insulin: control, 15.4 ± 1.0 days, PCOS-like females, 14.4 ± 0.5 days, control vs. PCOS-like: *p* ≤ 0.2). Overall duration of menstrual cycles remained the same (Figure 4; control females: diluent, 28.8 ± 3.1 days; insulin; 27.4 ± 1.0 days; PCOS-like females: diluent, 34.7 ± 2.6 days, insulin: 28.7 ± 1.0 days).

### 2.5. Ovarian Androgen Stimulation Test (at ~3–5 Months of Treatment)

There was no evidence of insulin-induced ovarian hyperandrogenism in either female group following an IM injection of 200 IU recombinant human chorionic gonadotropin (hCG) given during the 3rd to 5th early follicular phase or 30 day anovulatory period of treatment. Serum levels of 17αOHP, androstenedione, estradiol and progesterone increased (*p*-values ≤ 0.05) and peaked 24 h after hCG injection (Figure 4), but serum values and the degree of hCG-induced increase did not differ due to insulin treatment or female group. There were also no insulin treatment effects on steroid hormone ratios (Table 5) indicating relatively similar LH/CG receptor driven ovarian androgen biosynthesis (androstenedione to DHEA ratio), progesterone biosynthesis (progesterone to estradiol ratio) and aromatization of testosterone to estradiol (estradiol to testosterone ratio) in control compared to PCOS-like monkeys, regardless of treatment.

### 2.6. Gonadotropin-Releasing Hormone (GnRH) Test of Pituitary Gonadotropin Release

Insulin treatment did not alter GnRH-induced increases in circulating luteinizing hormone (LH) and follicle-stimulating hormone (FSH) in either control or PCOS-like monkeys in comparison to diluent treatment at 4–6 months following treatment onset. LH and FSH levels were increased and reached their peak values at 10 min after 20 µg GnRH intravenous injection in both female groups (FSH: *p* ≤ 0.03, LH: *p* ≤ 0.01; Table 6). There were no GnRH- or treatment-induced changes in the LH:FSH ratio in either female group (Table 6).

## 3. Discussion

Insulin acts through its cognate receptor in the ovary to modulate ovarian steroidogenesis, possibly acting synergistically with FSH and LH [82,83,84]. Intra-follicular insulin concentrations also reflect circulating insulin levels in both women [82] and rhesus monkeys [85]. It was therefore surprising that, despite ~2-fold elevations of circulating insulin levels in both PCOS-like and control adult female monkeys for 6–7 months and exceeding previously found insulin levels associated with anovulation in PCOS-like monkeys with high BMI [61], experimentally induced chronic hyperinsulinemia failed to enhance or induce PCOS-like reproductive or metabolic traits in either female monkey group. In fact, contrary to expectations, the lengthy follicular phase duration of PCOS-like females was normalized by insulin treatment, thus improving rather than impairing, ovulatory menstrual function in PCOS-like monkeys generated by gestational testosterone excess. In further contrast to our expectations, there was no insulin-induced ovarian hyperandrogenic response to an injection of hCG, and gonadotropin responses to exogenous GnRH remained typical. Hyperinsulinemia, alone, in the absence of pathological metabolic sequelae such as lipotoxicity [9], may thus not play a predominant role in the mechanism inducing PCOS-like traits in our non-human primate model of PCOS. Such a concept would be consistent with findings from the previous experiment employing PCOS-like monkeys and pioglitazone, an insulin-sensitizer [86]. Those findings suggested that consequences of insulin resistance (impaired insulin action, increased adiposity and lipotoxicity) rather than high circulating levels of insulin may be related to the mechanism of anovulation in PCOS-like monkeys [87]. The current insulin study also indicates that normal female monkeys cannot be converted to PCOS pathophysiology simply by inducing chronic elevations in their circulating insulin levels for 6–7 months.

The lack of expected reproductive impairments did not appear due to diminished bioactivity of administered insulin. Mean circulating glucose levels remained significantly reduced at ~93% of their placebo treatment values in both female groups and serum from only one control female exhibited notable specific binding of human insulin. In addition, our low incidence of hypoglycemia may be due to incremental increases in insulin administered (commencing at daily s.c. injections of 2.5U insulin) and intact counter-regulatory compensatory responses in these animals. Twice-daily glucose monitoring and avoidance of sudden dosage increases enabled stable maintenance of these subjects. Consistent with our findings in this monkey study, incremental increases in exogenous basal insulin therapy in humans with T2D without additional postprandial insulin administration, but with accompanying daily glucose monitoring, achieve clinically acceptable glycemic targets while mostly eliminating hypoglycemic episodes [55,74].

In vitro studies using human tissue indicate that insulin augments P450c17 activity in ovarian theca cells through PI3-kinase instead of MAPK pathways [41,83]. Previous attempts, however, to employ exogenous insulin to induce hyperandrogenism in normal or PCOS women [88,89] have not been particularly successful. Either serum testosterone levels were not elevated [88,89,90] or serum androstenedione levels were elevated in only PCOS [89] or normal [91] women.

As is possible in the present study, elevated levels of insulin may act on the ovary through more than one insulin receptor-mediated signaling pathway [82,90,91,92,93]. Similar inabilities to induce ovarian hyperandrogenism, however, have arisen when exogenous insulin-like growth factor-1 (IGF-1) was administered. The insulin receptor is homologous to the IGF-1 receptor [94,95], and IGF-1 binds to the IGF-1 receptor, hybrid insulin-IGF-1 receptors, and to the insulin receptor to a lesser extent [96,97]. IGF-1 receptors are expressed in rhesus monkey ovaries [98], and IGF-1 modulates ovarian follicle function [99]. Chronic (22 days) administration of excessive doses of recombinant human IGF-1 (240 micrograms/kg/day) to normal, adult female rhesus monkeys failed to induce ovarian hyperandrogenism [100]. Interestingly, in perhaps an analogous finding to this study, prepubertal female rhesus monkeys treated daily with exogenous IGF-1 (110 micrograms/kg/day) for ~18 months (without clamping of their endogenous gonadotropin secretion), exhibited first ovulation at a younger age than controls [101]. This may have been related to IGF-1-mediated diminished ability of estrogen to suppress LH secretion in females [100,102], resulting in elevated circulating LH levels [103]. In contrast, in four out of six women with primary growth hormone resistance (Laron syndrome), chronic treatment (many months) with excessive doses (120–150 micrograms/kg/day) of recombinant human IGF-1, resulted in hyperandrogenism [104], perhaps reflecting a supraphysiological dose of IGF-I.

In the context of chronic exogenous hyperinsulinism, T1D women exhibit increased incidence of hyperandrogenic disorders, including PCOS, compared to normal, non-diabetic women [59]. Women with T1D require daily insulin treatment to regulate their circulating glucose levels due to autoimmune or other destruction of pancreatic beta cells and their inability to synthesize insulin. Interestingly, Escobar-Morreale and colleagues [59] did not find an association between women receiving higher insulin doses and increased incidence of hyperandrogenism and PCOS. In a more recent paper, however, Thong and colleagues [39] have shown an association between increased use of intensive insulin therapy and increased prevalence of PCOS in women with T1D. Nevertheless, when comparing women with PCOS who did or did not exhibit T1D [105], PCOS women with T1D were found to have normal circulating levels of antimullerian hormone (AMH), estradiol and sex hormone binding globulin (SHBG), a normal LH/FSH ratio, and twice the incidence of regular menstrual cycles than PCOS women without T1D. Taken together, the results from these clinical studies suggest that chronic insulin treatment can mimic the hyperandrogenism, anovulation and ovarian morphology of PCOS, but through different pathophysiological mechanisms than PCOS.

The unexpected finding of insulin-induced improvement in ovulatory menstrual cycle function in PCOS-like female monkeys may reflect the well-documented action of insulin as a co-gonadotropin [22,41,83,92], possibly acting through insulin receptors on ovarian follicle cumulus and mural granulosa cells [82], as well as theca cells [41]. While no enhancement of ovarian theca cell responses to an hCG injection or gonadotropin responses to a GnRH injection were detected in the present study, injected exogenous insulin may well have synergized with endogenous LH and FSH to improve ovarian follicle granulosa function and follicular development [4,92,106,107]. This interpretation of our results is strengthened by the cross-over design of our study, removing any confound of increasing age that can lead to a degree of normalization of ovulatory menstrual cycles in women with PCOS [108].

Experimentally controlled, chronic insulin treatment (22 days) of ovulatory female rats promoted cystic ovarian morphology [109], but failed to induce anovulation or hyperandrogenism. Acute insulin treatment (6–16 h) of normal and PCOS women have yielded contradictory results. Circulating testosterone levels decreased in two studies [88,89], while circulating androstenedione levels increased in two other studies [89,91]. Exogenous insulin, nevertheless, has intra-ovarian follicle access to enable ovarian physiological changes in female rhesus monkeys, since both insulin receptor isoforms exist in the ovary [82], and intra-follicular insulin levels reflect circulating levels in both women [82] and female rhesus monkeys [85] undergoing ovarian hyperstimulation for in vitro fertilization (IVF).

Based on these unexpected findings in the present study, a revised understanding of pathophysiological mechanisms underlying anovulation and ovarian hyperandrogenism in PCOS-like female monkeys is suggested. Intensive insulin treatment may sufficiently compensate for impaired insulin action, thus providing effective co-gonadotropin support for steroidogenic and other functions with PCOS-like monkey ovaries, analogous to the unexpected effects of intensive (high dose) exogenous insulin treatment overcoming insulin resistance with regard to glucoregulation in T2D humans (at least 6 months of treatment) [110,111]. If PCOS-like monkey theca and/or granulosa cells exhibit insulin resistance in terms of metabolic signaling, such as diminished postprandial insulin-mediated glucose uptake, diminished expression of FSH/LH receptors and relevant steroidogenic enzymes such as *CYP11A1*, *CYP17A1*, *HSB3B2* and *CYP19A1*, might occur and impair follicle selection. Such an outcome might be expected since insulin acts as a co-gonadotropin in ovarian follicles [41,42,90] to promote cell survival and proliferation through the activity of phosphoinositide 3-kinase (PI3-Kinase) and mitogen-activated protein kinase (MAPK), to stimulate theca cell androstenedione and testosterone production as substrate for granulosa cell aromatization in PCOS women [112], and to enhance ovarian inositol-mediated insulin and gonadotropin action (thus reducing ovarian insulin resistance), supporting FSH-driven estradiol release from granulosa cells [42,44].

Such re-interpretation of ovarian pathophysiology in PCOS-like monkeys, through proposed impairments in insulin signaling in theca, granulosa or stromal cells (or a combination of all three), would agree with in vivo studies in PCOS women showing enhanced, rather than diminished, ovarian steroidogenic responses to FSH following insulin sensitizer treatment [107,113,114]. The latter studies, however, differ from several others showing unimpaired [24,84,115,116] or enhanced [19] insulin action on ovarian steroidogenic function in PCOS women due to insulin sensitizer treatment. With regard to glucose metabolism, though, insulin resistance is clearly present in PCOS ovaries [19]. The apparent discrepancies between the different PCOS studies may reflect the difference between in vivo investigation (evidence for ovarian insulin resistance, e.g., [107]) and work performed in vitro (evidence for lack of ovarian insulin resistance, e.g., [84,115]), as well as whether or not lipotoxicity or dysglycemia are also present [9,13].

With regard to strengths of this study, we employed the most comprehensive animal model for PCOS, PCOS-like female rhesus monkeys exposed to gestational testosterone excess during early- to mid-gestation. We also used previously validated quantitative assessment of insulin-mediated glucoregulation to provide accurate estimates of whole-body insulin sensitivity and pancreatic beta cell compensation, in combination with endocrine dynamic testing (i.e., hCG and GnRH challenge tests) to assess ovarian hyperandrogenism and pituitary hypergonadotropism in adult female rhesus monkeys. Limitations include small numbers of monkeys per treatment group, in compliance with contemporary refinement and reduction practices for laboratory non-human primates, and the use of adult female rhesus macaques in their late reproductive, pre-menopausal years. PCOS-like monkeys in this study may thus be more typical of women with PCOS, but without accompanying dysglycemia and lipotoxity [79,86,117], exhibiting less pronounced hyperandrogenism and hypergonadotropism, and combined ovulatory with oligo-ovulatory females, as occurs when PCOS is diagnosed using Rotterdam 2003 criteria [3,4].

In summary, this study examined whether experimentally induced hyperinsulinemia could induce PCOS-like traits in control female monkeys, and amplify such traits in PCOS-like monkeys exhibiting mild symptomology. Our findings indicate that, unlike a previous study employing an insulin-sensitizing agent to lower circulating insulin levels and normalize ovulatory menstrual cycles in PCOS-like female monkeys [86], we found no evidence for a role of elevated circulating insulin levels in the mechanism of PCOS pathogenesis. Our findings argue against a direct causal relationship between hyperinsulinemia alone and PCOS pathogenesis in female non-human primates, and likely, in women, as also proposed in studies of women with PCOS [13] and T1D or T2D [39,110,111]. Whether our findings indicate a basic pathophysiological difference between female rhesus monkeys and women is possible, but unlikely, since PCOS-like monkeys emulate traits required for diagnosis of PCOS in women [62,63], generated by experimental design or identified as naturally occurring [65]. As PCOS-like monkeys also demonstrate a large number of additional reproductive and metabolic traits associated with PCOS [62,63,65,118], and exhibit improved ovulatory menstrual cyclicity when treated with an insulin-sensitizing agent similar to women with PCOS [85], the present findings raise doubts about a simple, direct relationship between hyperinsulinemia, anovulation, hyperandrogenism and weight gain in women with PCOS.

## 4. Materials and Methods

### 4.1. Animals

A total of nine, captive-born adult female rhesus monkeys (*Macaca mulatta*), housed at the Wisconsin National Primate Research Center (WNPRC), were used in this study. Monkeys were maintained in accordance with routine care, management and assessment protocols [119]. Each monkey was fed once daily with a meal of 16–30 biscuits (approximately 96–180 g) of Purina Monkey Chow (Ralston Purina, Inc., St. Louis, MO; product # 5038) at ~0700–1000 h, within ~1 h of daily treatment (see below). The meal was supplemented with 1–2 pieces of fresh fruit, vegetables and/or bread at ~1500 h. The number of biscuits given was adjusted so that at least 1–3 biscuits were found when all remaining food in an animal’s cage was removed between ~1700 h and 1800 h. The Institutional Animal Care and Use Committee of the University of Wisconsin-Madison approved all the procedures used in this study. Animal maintenance was in accordance with the recommendations of the Guide for the Care and Use of Laboratory Animals and Animal Welfare Act with its subsequent amendments.

At baseline, the four control and five PCOS-like female monkeys used in this study were similar in age (control: 17.3 ± 1.0, PCOS-like: 20.7 ± 0.7, years), body weight (control: 8.8 ± 1.0, PCOS-like: 8.3 ± 0.4, kg), and body mass index (BMI) (control: 36.8 ± 3.6, PCOS-like: 36.5 ± 0.9, kg/m^2^) (age: *p* = 0.07, weight: *p* = 0.59, BMI: *p* = 0.92). The pregnant dams of PCOS-like monkeys received subcutaneous injections of 10 mg testosterone propionate (TP) starting during early gestation (days 40–44), for 15–35 consecutive days (term: 165 days). Many organ systems undergo differentiation during this early gestational period in rhesus monkeys, including the ovaries, hypothalamus/pituitary, and pancreas (63). The PCOS-like females selected for this study exhibited minimal PCOS-like traits, typical for ~25% of PCOS-like monkeys [64,117]. Controls comprised four normal, regularly cycling female rhesus monkeys without gestational exposure to TP.

### 4.2. Experimental Design

A cross-over experimental design was employed utilizing all monkeys in each of the two arms of this study (insulin or diluent daily injections). In the first arm of this study, two control and two PCOS-like females underwent daily subcutaneous (s.c.) injections of humulin U ultralente insulin (Humulin^®^ U Ultralente^®^, recombinant human insulin; Eli Lilly and Co., Indianapolis, IN), using a 28 g insulin syringe, for 6–7 months during the first treatment year (Humulin U Ultralente, Eli Lilly, IN; PCOS-like: 7.8 ± 1.7, control: 9.0 ± 1.5 U/day), while an additional two control and three PCOS-like females received daily placebo (Sterile Diluent, Eli Lilly and Company, Indianapolis, IN) s.c. injections. The second arm of this study in the following year utilized the same monkeys, but treatments given to each monkey were reversed. Each study arm duration lasted 6 to 7 months and avoided the summer-associated oligomenorrhea typical for this seasonally breeding species [62].

Insulin therapy was progressively increased in all monkeys in order to exceed 694.5 pmol/L (100 μU/mL) basal circulating levels of insulin (morning fasted insulin levels). This treatment dose of insulin was chosen so as to exceed elevated circulating insulin levels (694.5 pmol/L) previously associated with anovulation in PCOS-like female monkeys [61]. Exceeding this insulin level would permit testing of our hypothesis that hyperinsulinemia is functionally implicated in the mechanism of anovulation in our non-human model for PCOS. To initiate therapy, daily s.c. injections of 2.5U insulin were administered for the first 7 days between approximately 0630 h and 0930 h, immediately before the main meal of the day. After 7 days, the insulin dose was increased by 2.5 U/day every 3–4 days until serum insulin values exceeded 694.5 pmol/L in each female (from blood samples taken immediately before daily injection, i.e., 24 h after the previous insulin injection). Blood samples were assayed for insulin each week until target blood levels of insulin were reached, and then approximately every two weeks thereafter to ensure that circulating insulin levels were maintained at over 694.5 pmol/L.

To monitor circulating glucose levels during either chronic insulin or diluent therapy, blood glucose levels were obtained twice daily: (1) immediately before daily insulin or placebo injections (morning fasted), and (2) at ~1500 h, immediately prior to the daily supplement of fruit or bread (as described above). Blood glucose values were determined by a glucose meter (OneTouch Ultra, LifeScan, Milpitas, CA, USA) from spot blood samples taken from a peripheral vein. The twice-daily glucose values were highly correlated (r^2^ = 0.93, *p* ≤ 0.01) with those determined from the same samples by the glucose oxidase method, a validated and well-established procedure for accurately determining circulating glucose levels in rhesus monkeys [120]. We had no target level for circulating glucose in this study since our main objective was to achieve hyperinsulinemia (>694.5 pmol/L) in non-diabetic monkeys; rather, our twice-daily checks on circulating glucose were to ensure rapid detection of and response to occasional episodes of hypoglycemia. Hypoglycemic monkeys received a 5 mL intravenous injection of 5% dextrose solution and were fed an orange cut into segments.

During both treatment phases, metabolic and reproductive observations were recorded throughout the 6–7 months of each study arm, and a series of endocrine or glucoregulatory challenge tests were performed during the early follicular phase of menstrual cycles (cycle days 2–6) or anovulatory 30 day periods were performed in each of the two study arms. An ovarian androgen stimulation test was performed during the 2nd–3rd menstrual cycle or anovulatory 30 day period from the onset of treatment (~2–3 months following study onset); a frequently sampled, intravenous glucose tolerance test (FSIGTT) to assess insulin sensitivity was carried out in the subsequent menstrual cycle or anovulatory 30 day period (~3–4 months from treatment onset); and a GnRH stimulation test was performed during one of the next two menstrual cycles or anovulatory 30 day periods (~4–6 months from treatment onset).

### 4.3. Metabolic Observations

#### 4.3.1. Body Weight, and Select Glucoregulatory, Lipid and Hepatic Parameters

Body weights, fasted insulin, glucose and c-peptide, as well as select blood lipids (fasted total cholesterol and triglycerides) and hepatic enzyme measures for liver inflammation or damage (aspartate transaminase (AST) and alanine transaminase (ALT)), were assessed during each study arm at baseline and on completion of each treatment. Glucose was measured by the glucose oxidase method [120]. Insulin and c-peptide were determined by RIA [120]. Intra- and inter-assay CVs for plasma/serum quality control preparation values were: glucose: 2.9% and 4.0%, insulin: 4.6% and 7.9%, c-peptide: 4.3% and 8.3%.

#### 4.3.2. Assessment of Serum Binding of Insulin after 6–7 Months of Exogenous Insulin Treatment

Insulin assay buffer solution, rhesus serum quality control samples obtained from WNPRC Assay Services and serum samples from individual PCOS-like and control females 6–7 months after treatment onset were incubated with ^125^I-radiolabeled insulin (~15,000 counts per minute) and with an additional 100ul of assay buffer solution for 24 h at 4 °C. Endogenous binding of insulin above that found with insulin assay buffer and rhesus quality control serum indicated specific serum binding of insulin, rendering a portion of it unavailable for biological action.

### 4.4. Metabolic Challenge Test

#### Frequently Sampled Intravenous Glucose Tolerance Test (FSIGTT) at 3–4 Months of Treatment

Each monkey underwent a single FSIGTT as previously described [120] and during the 3rd–4th menstrual cycle (the follicular phase) or 30 day anovulatory period. Briefly, after an overnight fast, each animal was anesthetized with ketamine hydrochloride (15 mg/kg, i.m.) and diazepam (1.25 mg/kg, i.m.). Supplemental ketamine was administered as appropriate to maintain anesthesia (5–10 mg/kg, i.m.). A catheter was placed into the vena cava through the saphenous vein for blood sampling (e.g., 33 samples over 195 min) and for administration of glucose (300 mg/kg at 0 min) and tolbutamide (5 mg/kg at 20 min). Serum glucose and insulin levels were determined from these blood samples. Insulin sensitivity (S_I_; the measure of the fraction of glucose cleared from the circulation per unit increase in insulin) and glucose effectiveness (S_g_; the measure of the ability of glucose to increase its own uptake and to suppress hepatic glucose production at basal insulin levels) were determined using the modified minimal model method [78]. Further measures derived from the FSIGTT were basal insulin (I_b_; mean of the four prechallenge plasma insulin values, −15, −10, −5, and −2 min), basal glucose (G_b_; average of the four prechallenge plasma glucose values), basal c-peptide (average of the four prechallenge plasma c-peptide values), acute insulin responses to glucose (AIR_g_; average elevation of posthepatic plasma insulin concentration above the baseline for the 2, 3, and 4 min samples), acute insulin response to tolbutamide (AIR_tol_; average elevation of posthepatic plasma insulin concentration above the baseline for the 22, 23, and 24 min samples), glucose disappearance rate (K_G_; slope of the log linear regression of plasma glucose concentration between 10 and 19 min), and disposition index (DI or ß-cell compensation index; product of S_I_ and AIR_g_ [120]). Cumulative areas-under-the-curve (AUC) for insulin (INS), glucose (Glu) and C-peptide (c-pep) above baseline, and at selected times following glucose infusion indicated by _(minutes)_, were determined by the trapezoidal rule [121] (e.g., AUC_INS (0–19 min)_, cumulative acute insulin response to glucose infusion; AUC_INS (22–180 min)_, cumulative insulin values following tolbutamide-induced pancreatic beta cell depolarization resulting in insulin release; AUC_INS (0–180 min)_, cumulative combined insulin values following glucose and tolbutamide infusions; AUC_Glu (0–19 min)_, cumulative acute glucose values following glucose infusion; AUC_Glu (22–180 min)_, cumulative glucose values following tolbutamide-induced pancreatic beta cell depolarization resulting in insulin release; AUC_Glu (0–180 min)_, cumulative combined glucose values following glucose and tolbutamide infusions; AUC_c-pep (0–180 min),_ cumulative combined c-peptide values following glucose and tolbutamide infusions).

### 4.5. Reproductive Observations

#### Menstrual Cycle Assessment

Each monkey underwent saphenous venipuncture three times per week between 0630 h and 0930 h and the resultant serum was assayed for progesterone. Since menstrual discharge was undetectable in approximately one-third of ovulatory PCOS-like female rhesus monkeys [62], progesterone data were used to assess menstrual cycle characteristics in addition to monitoring occurrence of menses. The day that serum progesterone levels exceeded 0.4 ng/mL was designated as the first day of a luteal phase, while the day that serum progesterone levels declined below 0.4 ng/mL was defined the start of the follicular phase [62]. Ovulatory menstrual cycles were identified by finding two serum progesterone levels above 1 ng/mL within 15 days prior to menses [62,119]. The following parameters were derived from progesterone data: (1) duration of the luteal phase (the days between the onset of a luteal phase to the next onset of a follicular phase), (2) duration of the follicular phase (the days between the onset of a follicular phase to the onset of a luteal phase), (3) duration of the menstrual cycle (the days between the onset of a follicular phase to the onset of the next follicular phase), (4) area under curve (AUC ((ng/mL) × day)) serum progesterone for each luteal phase (as determined using the trapezoidal rule [121]), and (5) number of menstrual cycles (the number of menstrual cycles recorded in the first 6 months of the insulin or placebo treatment periods).

### 4.6. Endocrine Challenge Tests

#### 4.6.1. Ovarian Androgen Stimulation Test at 3–5 Months of Treatment

In the 3rd to 5th 30 day period or menstrual cycle follicular phase on treatment, each monkey was injected IM with 200 IU recombinant human chorionic gonadotropin (hCG) (Serono Laboratories, Norwell, MA) during the early follicular phase or an anovulatory period. Blood samples (5 mL) were withdrawn at 0 h (0800 h day 1), 24 h (0800 h day 2), 48 h (0800 h day 3) and 72 h (0800 h day 4). Blood was centrifuged at ~6400× *g* for 10 min, and serum was stored at −20 °C prior to assay. Serum was assayed for progesterone, 17α-hydroxyprogesterone (17αOHP), dehydroepiandrosterone (DHEA), androstenedione, testosterone and estradiol to assess ovarian responses to LH/CG receptor stimulation. Dose of hCG, timing of blood samples and hormone assays employed were comparable to those used in diagnosing ovarian hyperandrogenism in women [122] and previously validated in PCOS-like and control female rhesus monkeys [63,123].

#### 4.6.2. GnRH Test at 4–6 Months of Treatment

A GnRH test was performed during the 4–6th 30 day period or menstrual cycle follicular phase of insulin or placebo treatment. Monkeys were anesthetized with an intramuscular (IM) injection of 15 mg/kg ketamine hydrochloride at around 0745 h. Then, a venous catheter (Polyethylene tubing, Intramedic TM, PE60, Becton Dickinson and Company, Sparks, MD, USA) was inserted through the saphenous vein and positioned in the inferior vena cava for the whole procedure. At ~0800 h, the monkey was infused IV through the catheter with 20 μg GnRH (0.5 mL, Sigma L-7134; Sigma Chemical Company, St. Louis, MO, USA [124]) and blood samples (3 ml) were withdrawn at 0, 2, 5, 10, 20, 30, 40, and 60 min after GnRH infusion. Blood was centrifuged at ~6400× *g* for 10 min, and serum stored at −40 °C until immunoactive LH and FSH were assayed.

### 4.7. Assay Procedures

All hormones were assayed in the WNPRC Hormone Assay Services Laboratory [62,125,126]. Assays for DHEA, androstenedione, testosterone, and estradiol were performed following diethyl ether extraction of serum and solvent fraction separation by celite chromatography. DHEA, estradiol, androstenedione [62], 17αOHP [123], LH, and FSH [62] determinations were assayed using RIAs. Progesterone and testosterone were assayed by enzyme-IAs (EIAs) [62]. Intra- and inter-assay CVs for quality control preparation (QC) values were: progesterone: 3.7% and 19.4%; 17αOHP: 5.6% and 9.8%; DHEA: 8.2% and 13.4%; androstenedione: 3.7% and 7.9%; testosterone 3.5% and 17.7%; estradiol: 9.3% and 20.0%; FSH: 3.2% and 4.0%; LH: 4.2% and 4.3%.

### 4.8. Statistical Analysis

Data were log_10_ transformed to achieve homogeneity of variance and to increase linearity when appropriate [127]. Data were analyzed using Proc GLM with repeated measures (SAS, version 8, SAS Institute Inc., Cary, NC, USA). When log_10_ transformation failed to normalize data, non-parametric tests were performed (SAS, Version 8, SAS Institute Inc., Cary, NC, USA). Paired t-tests were used to determine whether serum LH or FSH increased at 10 min after GnRH injection (0 min vs. 10 min for each animal). *p*-values of 0.05 or less were taken as significant and are all shown to two decimal places. Data were expressed as the mean ± SEM.

## Figures and Tables

**Table 1:** Morning fasted serum insulin and glucose values (mean ± SEM) between 630 h and 930 h, as well as afternoon glucose levels at ~1500 h, in control (*n* = 4), and PCOS-like (*n* = 5), adult female rhesus monkeys at baseline (0 months) and during 1–6 months of insulin and diluent treatments. Monkeys each received six months of insulin treatment followed or preceded by six months of diluent (vehicle) treatment as part of a cross-over experimental design.

Hormone Treatment	Time (Months) Following Study Onset						
Female Group	0	1	2	3	4	5	6
**Fasted insulin (pmol/L)**							
Diluent group							
Control	160 ± 12	333 ± 35	397 ± 30	374 ± 66	270 ± 25	215 ± 3	220 ± 4
PCOS-like	375 ± 71	305 ± 40	371 ± 92	410 ± 78	337 ± 48	220 ± 2	216 ± 3
Insulin group ^a^							
Control	182 ± 49	338 ± 106	557 ± 98	622 ± 130	841 ± 195	880 ± 115	890 ± 106
PCOS-like	267 ± 91	403 ± 105	442 ± 123	610 ± 102	793 ± 151	926 ± 52	996 ± 36
**Fasted glucose (mmol/L)**							
Diluent group							
Control	3.1 ± 0.1	3.1 ± 0.1	3.0 ± 0.1	3.0 ± 0.1	3.0 ± 0.1	3.2 ± 0.1	3.2 ± 0.1
PCOS-like	2.9 ± 0.1	3.2 ± 0.2	3.0 ± 0.2	3.1 ± 0.2	3.3 ± 0.2	3.4 ± 0.2	3.3 ± 0.2
Insulin group ^b^							
Control	2.9 ± 0.2	3.0 ± 0.1	2.8 ± 0.	2.8 ± 0.2	3.2 ± 0.1	3.2 ± 0.1	3.3 ± 0.1
PCOS-like	5.3 ± 1.5	5.1 ± 1.3	4.1 ± 0.8	4.6 ± 1.0	5.4 ± 1.3	5.1 ± 0.8	4.7 ± 0.9
**Afternoon glucose (mmol/L)**							
Diluent group							
Control	3.2 ± 0.3	3.0 ± 0.1	3.0 ± 0.1	3.1 ± 0.1	3.1 ± 0.1	3.3 ± 0.1	3.4 ± 0.1
PCOS-like	3.0 ± 0.1	3.3 ± 0.1	3.3 ± 0.2	3.3 ± 0.1	3.4 ± 0.2	3.4 ± 0.2	3.5 ± 0.2
Insulin group ^c^							
Control	3.2 ± 0.3	3.2 ± 0.3	3.1 ± 0.1	3.2 ± 0.1	3.3 ± 0.1	3.2 ± 0.1	3.4 ± 0.1
PCOS-like	5.7 ± 1.3	6.3 ± 2.2	6.0 ± 2.1	6.2 ± 2.3	5.8 ± 1.9	5.4 ± 0.2	5.5 ± 1.9

^a^*p* < 0.01 vs. diluent for PCOS-like and control females, as well as all time points, combined. ^b^ *p* < 0.01 vs. diluent, for PCOS-like and control females combined. ^c^ *p* < 0.01 vs. diluent in PCOS-like females alone (Bonferroni corrected for two comparisons).

**Table 2:** Mean ± SEM circulating serum insulin levels (pmol/L) over a single 24 h period following daily diluent or insulin injection at 0 h, immediately before the 1st daily feeding and well before the 2nd feeding of the day at ~1500 h (approximately 7–8 h after daily insulin injection). The assessment was made during the follicular phase of a menstrual cycle or anovulatory period at ~3–5 months following each treatment onset.

Female Treatment Groups	Hours 0 h	Following 2 h	Daily 4 h	Injection 24 h
Control diluent (*n* = 4)	373 ± 79	743 ± 240	961 ± 261	476 ± 110
Control insulin ^a^ (*n* = 4)	972 ± 321	672 ± 44	3966 ± 1340 ^b^	964 ± 244 ^c^
PCOS-like diluent (*n* = 5)	522 ± 193	925 ± 192	826 ± 214	517 ± 152
PCOS-like insulin ^a^ (*n* = 5)	760 ± 90	2125 ± 830	1345 ± 387 ^b^	927 ± 325 ^c^

^a^*p* < 0.01 vs. diluent (when control and PCOS-like female monkeys are combined); ^b^ *p* < 0.02 vs. 0 h and 2 h (when control and PCOS-like female monkeys are combined); ^c^ *p* < 0.01 vs. 4 h (when control and PCOS-like female monkeys are combined).

**Table 3:** Minimal model and additional parameters (mean ± SEM) derived from frequently sampled, IV glucose tolerance tests administered during the follicular phase of the 3rd–4th menstrual cycle or 30 day anovulatory period (~3–4 months) from treatment onset for each of the diluent and insulin treatment phases in control (*n* = 4) and PCOS-like (*n* = 5) female rhesus monkeys.

Parameters	Control Monkeys		PCOS-like Monkeys	
	Diluent	Insulin	Diluent	Insulin
G_b_ (mmol/L)	3.2 ± 0.2	3.6 ± 0.6	3.5 ± 0.2	2.2 ± 0.6
I_b_ (pmol/L)	294 ± 117	478 ± 139 ^a^	204 ± 117	557 ± 139 ^a^
Kg (%/min)	9.8 ± 1.5	4.9 ± 0.9	5.0 ± 1.5	4.4 ± 0.9
S_g_ (×10^−2^/min)	4.1 ± 1.1	5.4 ± 1.0	4.1 ± 1.1	4.8 ± 1.0
S_I_ (×10^−5^/min/pmol/L)	2.0 ± 1.8	1.6 ± 0.9	3.9 ± 1.8	2.2 ± 0.9
DI (×10^2^/min)	4.8 ± 2.3	3.0 ± 0.7 ^b^	4.8 ± 2.3	0.6 ± 0.7 ^b^
AIR_g_ (pmol/L)	251 ± 58	233 ± 48 ^c^	134 ± 58	37 ± 48 ^c^
AIR_tol_ (pmol/L)	276 ± 72	520 ± 117	242 ± 72	143 ± 58
AUC_INS (0–19)_ (pmol/L × 19 min × 10^3^)	38 ± 91	60 ± 11	27 ± 91 ^d^	18 ± 11 ^d^
AUC_INS (22–180)_ (pmol/L × 158 min × 10^3^)	84 ± 38	183 ± 58	63 ± 38	97 ± 58
AUC_INS (0–180)_ (pmol/L × 180 min × 10^3^)	123 ± 44	244 ± 69 ^e^	90 ± 44	115 ± 69 ^e^
AUC_GLU (0–19)_ (mmol/L × 19 min)	204 ± 11	237 ± 20	206 ± 11	211 ± 20
AUC_GLU (22–180)_ (mmol/L × 158 min)	503 ± 48	585 ± 100	547 ± 48	585 ± 100
AUC_GLU (0–180)_ (mmol/L × 180 min)	716 ± 50	819 ± 115	752 ± 50	796 ± 115
Basal C-peptide (nmol/L) × 10^−1^	1.9 ± 0.6	1.1 ± 1.4	1.6 ± 0.6	5.3 ± 1.4
AUC_c-pep (0–180)_ (nmol/L × 180 min)	33.3 ± 11.2	31.7 ± 2.7	29.3 ± 7.3	98.3 ± 29.0

^a^*p* < 0.01 vs. diluent, when control and PCOS-like female monkeys are combined; ^b^ *p* < 0.02 vs. diluent, when control and PCOS-like female monkeys are combined; ^c^ *p* < 0.02 vs. diluent, when control and PCOS-like female monkeys are combined; ^d^ *p* < 0.03 vs. control female monkeys, when diluent and insulin phases are combined; ^e^ *p* < 0.04 vs. diluent, when control and PCOS-like female monkeys are combined.

**Table 4:** Mean (± SEM) body weight, lipid and liver enzyme values at baseline and after 6–7 months (mo) of diluent or insulin treatment in adult female control (*n* = 4) and PCOS-like (*n* = 5) rhesus monkeys.

	Diluent Baseline	Diluent 6–7 mo	Insulin Baseline	Insulin 6–7 mo
Body weight Control (kg)	8.4 ± 0.6	8.8 ± 0.9	8.5 ± 0.3	9.0 ± 0.6
Body weight PCOS-like (kg)	8.8 ± 0.3	8.4 ± 0.4	9.1 ± 0.6	9.5 ± 0.5
Cholesterol Control (mg/mL)	150 ± 13	137 ± 4	172 ± 10	151 ± 7
Cholesterol PCOS-like (mg/mL)	157 ± 14	162 ± 14	116	140 ± 9
Triglyceride Control (mg/mL)	158 ± 55	55 ± 19	130 ± 36	235 ± 32
Triglyceride PCOS-like (mg/mL)	191 ± 28	88 ± 42	363	185 ± 95
AST Control (mU/mL)	31 ± 5	36 ± 10	42 ± 7	32 ± 14
AST PCOS-like (mU/mL)	25	22	29	42 ± 2
ALT Control (mU/mL)	33 ± 6	32 ± 4	51 ± 21	33 ± 7
ALT PCOS-like (mU/mL)	43	27	9	20 ± 6

Diluent and insulin baseline: within 1 month before the onset of treatment; AST: aspartate aminotransferase; ALT: alanine aminotransferase; AST and ALT values without SEM represent mean values of only two monkeys per treatment group due to lost samples. SI units conversion: cholesterol × 0.0259 mmol/L; triglyceride × 0.01129 mmol/L. There were no significant differences between female group or treatments.

**Table 5:** Mean ± SEM circulating ratios of selected steroid hormones in adult female control (*n* = 4) and PCOS-like (*n* = 5) monkeys during an ovarian androgen stimulation test (intramuscular injection of 200IU human chorionic gonadotropin at 0 min). Monkeys each received six months of insulin treatment followed or preceded by six months of diluent (vehicle) treatment as part of a cross-over experimental design. The ovarian androgen stimulation test was administered 3–5 months after each treatment onset during an early- to mid-follicular phase of a menstrual cycle or an anovulatory period.

Hormone Ratio Treatment	Time (Hours) Following 200IU Intramuscular Injection of rhCG			
Female Group	0	24	48	72
**Androstenedione:Dehydroepiandrosterone**				
Diluent				
Control	6.8 ± 1.6	6.5 ± 2.9	7.7 ± 2.1	17.4 ± 11.0
PCOS-like	2.7 ± 0.2	6.3 ± 1.4	4.5 ± 0.9	1.8 ± 0.5
Insulin				
Control	2.4 ± 0.8	3.5 ± 1.3	2.8 ± 0.4	2.3 ± 0.5
PCOS-like	7.2 ± 2.1	6.6 ± 2.6	7.8 ± 2.2	5.6 ± 2.4
**Testosterone:Androstenedione × 10^−2^**				
Diluent				
Control	1.6 ± 0.2	1.9 ± 0.5	2.3 ± 0.6	2.1 ± 0.3
PCOS-like	1.5 ± 0.2	1.2 ± 0.1	1.6 ± 0.4	1.4 ± 0.2
Insulin				
Control	1.7 ± 0.5	1.1 ± 0.2	1.1 ± 0.2	1.1 ± 0.1
PCOS-like	1.0 ± 0.4	0.7 ± 0.4	0.9 ± 0.5	0.8 ± 0.4
**Testosterone:Estradiol**				
Diluent				
Control	15.4 ± 2.9	11.3 ± 3.2	27.8 ± 12.5	19.8 ± 2.7
PCOS-like	12.4 ± 6.5	7.3 ± 1.5	14.3 ± 1.3	12.3 ± 1.1
Insulin				
Control	18.7 ± 5.9	8.3 ± 2.6	14.5 ± 5.5	11.9 ± 3.5
PCOS-like	22.3 ± 6.8	15.5 ± 8.3	19.1 ± 6.6	25.3 ± 10.9
**Estradiol:Progesterone × 10^−2^**				
Diluent				
Control	23.5 ± 11.5	2.7 ± 0.9	10.0 ± 8.3	8.0 ± 4.4
PCOS-like	38.9 ± 17.3	7.9 ± 2.5	4.5 ± 1.0	4.0 ± 0.3
Insulin				
Control	11.3 ± 6.4	4.3 ± 1.4	3.0 ± 0.9	5.9 ± 1.5
PCOS-like	4.3 ± 1.3	4.4 ± 1.4	3.6 ± 1.0	3.3 ± 1.6

There were no significant differences between female group or treatments.

**Table 6:** Mean ± SEM circulating LH and FSH levels, and LH:FSH ratio in adult female control (*n* = 4) and PCOS-like (*n* = 5) monkeys following 20 μg gonadotropin-releasing hormone (GnRH) intravenous injection at 0 min. Monkeys each received 6–7 months of insulin treatment followed or preceded by 6–7 months of diluent (vehicle) treatment as part of a cross-over experimental design. GnRH was administered 4–6 months after each treatment onset during an early- to mid-follicular phase of a menstrual cycle or an anovulatory period.

Hormone Treatment	Time (Minutes) Following GnRH Injection							
Female Group	0	2	5	10	20	30	40	60
**LH (ng/mL)**								
Diluent								
Control	1.2 ± 0.4 ^a^	1.2 ± 0.3	1.3 ± 0.4	1.4 ± 0.4 ^c^	1.3 ± 0.4	1.4 ± 0.4	1.3 ± 0.4	1.3 ± 0.4
PCOS-like	1.3 ± 0.4 ^a^	2.3 ± 0.8	2.7 ± 1.0	2.7 ± 1.0 ^c^	2.8 ± 0.9	2.6 ± 0.8	2.5 ± 0.8	2.4 ± 1.0
Insulin								
Control	1.1 ± 0.3 ^a^	1.1 ± 0.3	1.4 ± 0.4	1.5 ± 0.3 ^c^	1.6 ± 0.4	1.6 ± 0.4	1.7 ± 0.5	1.5 ± 0.4
PCOS-like	1.0 ± 0.2 ^a^	1.3 ± 0.1	1.6 ± 0.1	1.6 ± 0.1 ^c^	1.6 ± 0.1	1.7 ± 0.1	1.7 ± 0.1	1.6 ± 0.1
**FSH (ng/mL)**								
Diluent								
Control	1.8 ± 0.3 ^b^	1.6 ± 0.2	1.9 ± 0.4	1.7 ± 0.4 ^c^	1.8 ± 0.4	1.7 ± 0.3	1.8 ± 0.5	1.8 ± 0.4
PCOS-like	3.9 ± 1.3 ^b^	4.5 ± 1.5	4.5 ± 1.5	4.8 ± 1.6 ^c^	5.4 ± 1.6	5.2 ± 1.4	5.3 ± 1.5	5.3 ± 1.5
Insulin								
Control	2.8 ± 1.6 ^b^	2.8 ± 1.5	2.7 ± 1.5	2.8 ± 1.4 ^c^	2.7 ± 1.0	2.7 ± 1.1	2.9 ± 1.5	2.9 ± 1.4
PCOS-like	3.0 ± 1.0 ^b^	2.9 ± 0.8	3.3 ± 1.0	3.2 ± 0.8 ^c^	3.3 ± 0.8	3.2 ± 0.6	3.5 ± 0.9	3.5 ± 0.9
**LH:FSH ratio**								
Diluent								
Control	0.8 ± 0.2	0.8 ± 0.2	0.7 ± 0.2	0.8 ± 0.2	0.8 ± 0.2	0.8 ± 0.2	0.8 ± 0.2	0.7 ± 0.2
PCOS-like	0.4 ± 0.1	0.5 ± 0.1	0.6 ± 0.1	0.6 ± 0.1	0.5 ± 0.1	0.5 ± 0.1	0.5 ± 0.1	0.4 ± 0.1
Insulin								
Control	0.5 ± 0.1	0.5 ± 0.1	0.7 ± 0.2	0.7 ± 0.1	0.7 ± 0.1	0.7 ± 0.1	0.8 ± 0.1	0.6 ± 0.1
PCOS-like	0.6 ± 0.2	0.7 ± 0.3	0.8 ± 0.3	0.7 ± 0.3	0.7 ± 0.2	0.7 ± 0.2	0.7 ± 0.2	0.6 ± 0.2

^a^*p* < 0.03 vs. 2–60 min, when control and PCOS-like female groups, as well as diluent and insulin treatments, are combined. ^b^ *p* < 0.01 vs. 2–60 min, when control and PCOS-like female groups, as well as diluent and insulin treatments, are combined. ^c^ *p* < 0.03 vs. 0 min, when control and PCOS-like female groups, as well as diluent and insulin treatments, are combined.

## Data Availability

The data supporting the reported results can be obtained from the corresponding author on request.
